# Metal-Oxide Heterojunction: From Material Process to Neuromorphic Applications

**DOI:** 10.3390/s23249779

**Published:** 2023-12-12

**Authors:** Yu Diao, Yaoxuan Zhang, Yanran Li, Jie Jiang

**Affiliations:** Hunan Key Laboratory of Nanophotonics and Devices, School of Physics, Central South University, 932 South Lushan Road, Changsha 410083, China

**Keywords:** metal oxide, heterojunction, memristor, transistor, neuromorphic applications

## Abstract

As technologies like the Internet, artificial intelligence, and big data evolve at a rapid pace, computer architecture is transitioning from compute-intensive to memory-intensive. However, traditional von Neumann architectures encounter bottlenecks in addressing modern computational challenges. The emulation of the behaviors of a synapse at the device level by ionic/electronic devices has shown promising potential in future neural-inspired and compact artificial intelligence systems. To address these issues, this review thoroughly investigates the recent progress in metal-oxide heterostructures for neuromorphic applications. These heterostructures not only offer low power consumption and high stability but also possess optimized electrical characteristics via interface engineering. The paper first outlines various synthesis methods for metal oxides and then summarizes the neuromorphic devices using these materials and their heterostructures. More importantly, we review the emerging multifunctional applications, including neuromorphic vision, touch, and pain systems. Finally, we summarize the future prospects of neuromorphic devices with metal-oxide heterostructures and list the current challenges while offering potential solutions. This review provides insights into the design and construction of metal-oxide devices and their applications for neuromorphic systems.

## 1. Introduction

Since the invention of the computer, technological progress has led to continuous improvements in computer performance and device function. Especially in recent years, the rapid technological developments for the Internet of Things and artificial intelligence (AI) have significantly increased the requirements for computer data processing [1,2,3]. Traditional von Neumann architectures could successfully solve the structural problem [4], but they encounter substantial challenges in contemporary computing, which are named the von Neumann bottleneck. At present, there are growing developments of new algorithms, such as artificial neural networks and deep learning [5,6]. Meanwhile, since the complementary metal-oxide semiconductor (CMOS) transistor may have reached its limit in terms of size, Moore’s law may begin to break down [7]. In contrast, the human brain, with a high parallelism, has a low power of 20 W because the average synaptic power consumption is only about 10 fJ [8,9,10]. Therefore, brain-inspired neuromorphic devices have attracted increasing interest as a potential solution for breaking the von Neumann bottleneck [11].

At the same time, metal-oxide materials may provide an ideal platform for the development of multifunctional neuromorphic devices due to their low power consumption, high stability, versatility, low cost, etc. [12,13,14]. Moreover, as basic building blocks, metal-oxide heterojunctions have an intriguing performance due to their higher charge mobility, lower leakage current, and faster response time ascribed to interfacial states and band bending [15,16,17,18]. Such heterojunctions allow the combination of different types of metal oxides to achieve more functionalities and better performances. Therefore, neuromorphic devices based on metal-oxide heterojunctions may provide a great opportunity for next-generation computing technologies.

**Figure 1:**
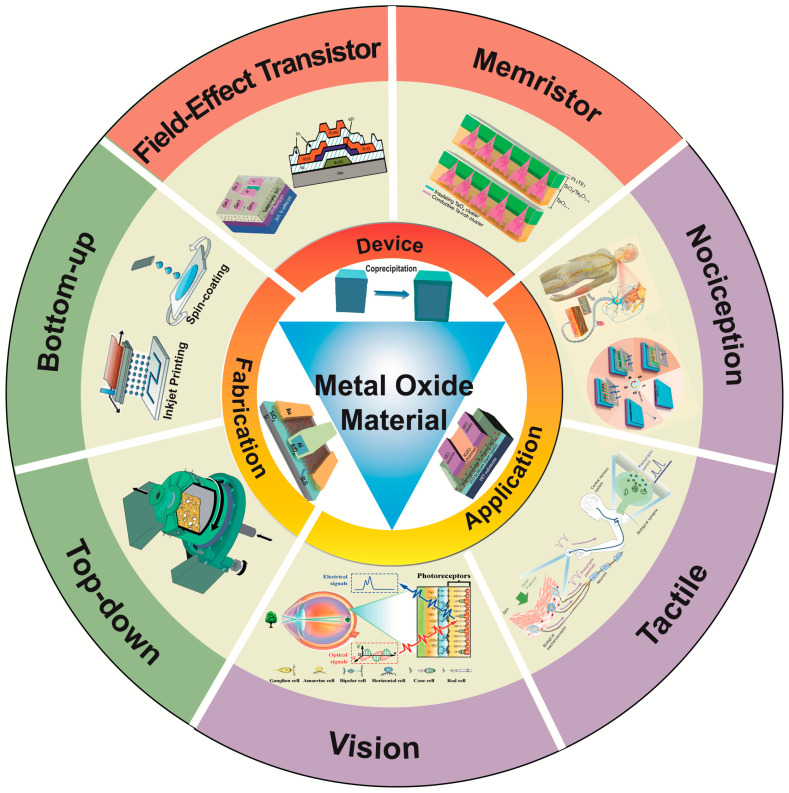
An overview of metal-oxide materials for neuromorphic applications. Reprinted with permission from [19]. Copyright 2023, IOP Publishing on behalf of the Chinese Physical Society. Reprinted with permission from [20]. Copyright 2021, Journal of Semiconductors. Reprinted with permission from [21]. Copyright 2020, American Chemical Society. Reprinted with permission from [22]. Copyright 1972, Royal Society of Chemistry. Reprinted with permission from [23]. Copyright 2021, Springer Nature. Reprinted with permission from [24]. Copyright 2019, John Wiley and Sons. Reprinted with permission from [25]. Copyright 2019, John Wiley and Sons. Reprinted with per-mission from [26]. Copyright 2021, John Wiley and Sons.

In this review, we first describe the various process methods of metal oxides and their heterojunctions. Then, we summarize the various devices and multifunctional neuromorphic applications based on metal-oxide materials and heterostructures, as shown in Figure 1. Finally, the key challenges and future directions are discussed for these kinds of neuromorphic devices. This review can provide a good understanding and guidance for entering the field of neuromorphic devices based on metal-oxide heterojunctions.

## 2. Metal-Oxide Heterojunction

### 2.1. Preparation of Metal-Oxide Materials

The synthesis and preparation of metal-oxide materials can be classified into two main categories: bottom-up and top-down approaches.

#### 2.1.1. Bottom-Up Approaches

**Figure 2:**
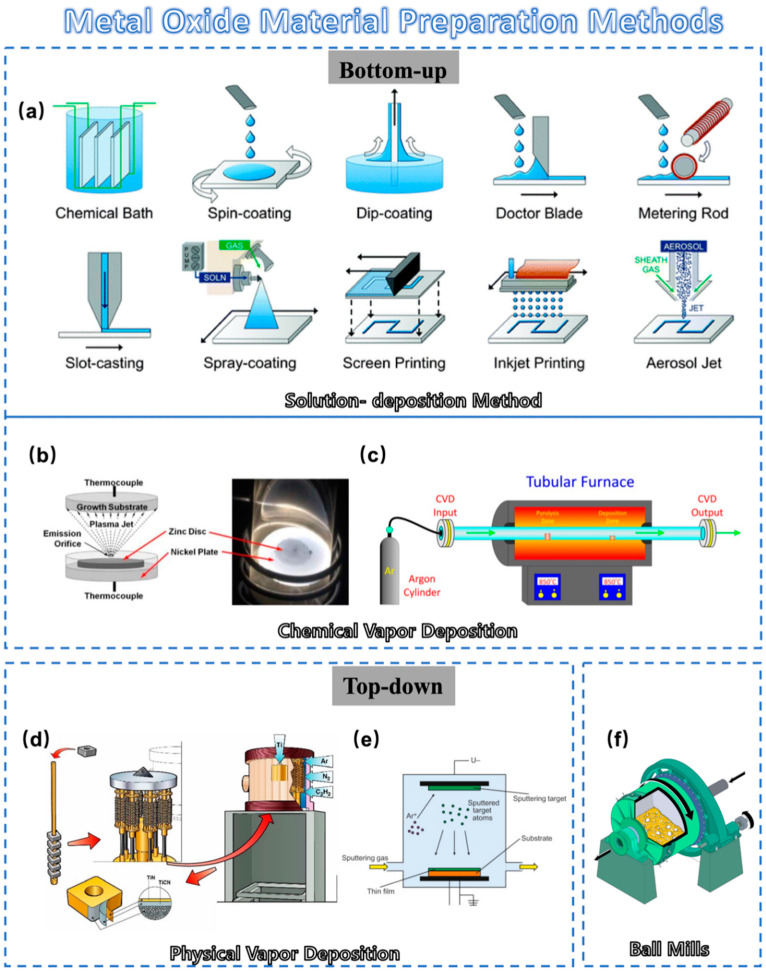
Schematic diagram of metal-oxide material preparation method: (**a**) Schematic of various solution-deposition processes: direct material growth without additional processing and liquid coating with additional processing to remove the solvent. Reprinted with permission from [22]. Copyright 1972, Royal Society of Chemistry. (**b**) Schematic cross-section of a thermal plasma chemical vapor deposition (CVD) system used to deposit ZnO nanocrystal thin films. Reprinted with permission from [34]. Copyright 2011, Springer Nature. (**c**) A scheme showing the setup used to grow carbon nanotubes inside nanoporous anodic alumina templates by CVD [35]. (**d**) Schematic diagram of conventional physical vapor deposition (PVD) process [36]. (**e**) Schematic illustration of the PVD process. Reprinted with permission from [37]. Copyright 2023, Elsevier. (**f**) A section cut-through of a ball mill. Reprinted with permission from [23]. Copyright 2021, Springer Nature.

- **Solution-Deposition Method**: Solution-deposition methods include a variety of methods (Figure 2a). Solution techniques allow for the deposition of films at atmospheric pressure with minimal equipment cost. Scalable deposition methods that allow for uniform, large-area coverage are important for high-throughput industrial applications [22]. We highlight the sol–gel method and inkjet printing method in this section. The latter method mainly involves transforming the desired precursor material into an inkjet state and then distributing it for printing in the same way as other solution-deposition methods. A new kind of fully inkjet-printed InP/ZnSe QD/SnO_2_ heterostructure thin film transistor (TFT) was fabricated with a bottom-gate, top-contact configuration by Liang et al. [27]. The fully printed device on a glass substrate exhibited a high optical transparency in the visible spectrum. This chemical method allows the formation of metal oxides using a colloidal solution (sol) approach. This method can well control composition and structure at the nanoscale. However, this process is always slow and often requires post-synthesis calcination [28].
- **Chemical Vapor Deposition**: Chemical vapor deposition is a process in which the substrate is exposed to one or more volatile precursors, and then reaction and decomposition are performed on the substrate surface to create the desired thin film. In typical CVD (Figure 2b), precursor gases are fed into the reaction chamber at ambient temperature. When they pass or come in contact with the heated substrate, they react or decompose to form a solid phase and then are deposited on the substrate. As shown in Figure 2c, the substrate temperature is very important and can affect the reactions that will take place. Compared to sol–gel methods, it typically offers a high degree of compositional and crystal structure control for precise material design. As a result, CVD has attracted growing interest in the semiconductor industry due to its large-area growth ability [29,30,31].
- **Electrodeposition**: Electrodeposition is a method for depositing ions from a solution onto an electrode’s surface through an electrochemical reaction [32]. For example, Yin et al. easily prepared Cu_2_O thin films using the electrodeposition method, thereby creating P-type Cu-based metal-oxide materials with excellent photoelectrochemical water splitting capability [32]. Unlike CVD, electrodeposition typically does not require high-temperature treatment or hazardous gases, making it environment-friendly. Moreover, it is a cost-effective way as it does not necessitate high-vacuum equipment or expensive precursors. These advantages make it an emerging favorite for the electronic materials [32,33].

#### 2.1.2. Top-Down Approaches

- **Mechanical Milling**: Mechanical milling is a very traditional and simple method of making metal-oxide powders. In this approach, metal-oxide powders are directly generated by mechanically grinding the bulk materials using a ball mill, as shown in Figure 2f. The electrical properties of metal oxides can easily be altered by adding other materials such as metals in this traditional method, as reported by Mikio et al. [38]. Using this method, Ag particles were added to Na_x_Co_2_O_4_ thermoelectric oxide. A mechanical milling process was included for uniform dispersion of Ag particles in the Na_x_Co_2_O_4_ matrix phase. Mechanical milling is also effective in reducing grain size, which is expected to affect the electrical resistivity and thermal conductivity of the product. However, it often lacks the capability of precise control over particle size distribution [38].
- **Physical Vapor Deposition**: As one of the most common processes on the market (Figure 2d), PVD offers distinct advantages compared to other methods for preparing metal-oxide materials [36]. It excels in its precision at the micro- and nanoscale, resulting in high-quality, uniformly dense films (Figure 2e). Moreover, PVD operates at lower temperatures, reducing the risk of thermal stress [39]. PVD films are high-purity, environment-friendly, and compatible with most materials and substrates. These attributes render PVD highly sought after in electronic/optical coatings and material enhancement [40,41]. PVD methods for preparing metal-oxide materials encompass magnetron sputtering, laser deposition, ion beam deposition, molecular beam epitaxy, thermal evaporation, etc. These techniques play an important role in preparing metal-oxide films with desired structural properties [42,43,44,45,46,47].

### 2.2. The Advantages of Heterojunctions

#### 2.2.1. Types of Heterojunctions

We classify heterojunctions into all-organic heterojunctions, mixed organic–inorganic heterojunctions, and all-inorganic heterojunctions based on the type of material. All-organic heterojunctions usually have better processability and bendability [48], and substrate selection and growth techniques play a large role in achieving the desired material properties [49]. With high stability and high electron mobility, all-inorganic heterojunctions allow for device performance and are less flexible. These materials usually require high temperatures or complex preparation processes. Organic–inorganic hybrid heterojunctions combine the processability of organic materials with the high stability and high electron mobility of inorganic materials [50]. This structure allows for a better balance of electron and hole transport, improving the performance of electronic devices. However, the combination of such heterojunctions often requires a high degree of matching [51]. The choice of which heterojunction to use depends largely on the needs of the particular application, such as flexibility, stability, processing difficulty, and cost. Due to the presence of some micro/nano interactions such as surface and interface engineering, quantum confinement effects, strain engineering, chemical interactions, and doping [52,53,54,55,56], all kinds of heterojunctions have something in common; that is, the formation of heterojunctions allows the material properties to be improved dramatically, which is the basis of why heterojunctions involving metal oxides can be used in a wide range of applications, and this provides enlightenment for metal-oxide heterojunctions in neuromorphic applications.

#### 2.2.2. Applications of Heterojunctions

We have already introduced the superior properties of heterojunctions, and when metal-oxide materials are involved in heterojunctions, they often have their own unique advantages. In this section, we will introduce the properties and applications of heterojunctions with the participation of metal oxides in some common areas.

- Electrode materials for supercapacitors (SCs)

In recent years, transition-metal oxides (TMOs) have often been considered as the main cathode material for supercapacitors because of their versatile redox reactions, high theoretical capacitance, and economic feasibility [57,58]. However, TMOs always possess relatively low electrical conductivity, which leads to poor electrochemical performance, such as low specific capacitance, rate performance, and cycling stability [59].

**Figure 3:**
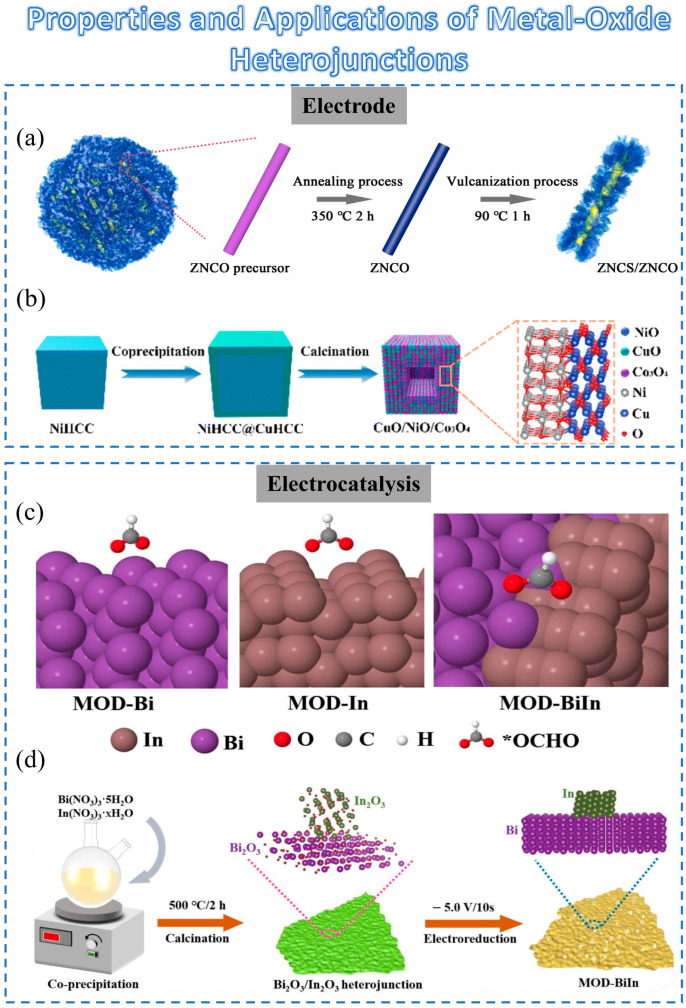
Schematic diagram of the preparation method for metal-oxide heterojunction and its applications: (**a**) Schematic formation process of ZNCS/ZNCO heterostructure composites. Reprinted with permission from [60]. Copyright 2023, Elsevier. (**b**) Schematic diagram of the procedure for a hollow CuO/NiO/Co_3_O_4_ heterostructure. Reprinted with permission from [61]. Copyright 2003, Royal Society of Chemistry. (**c**) The illustration of the fabrication process for Bi_2_O_3_/In_2_O_3_ heterojunction (MOD-BiIn) hybrid material. (**d**) The overview schematic of *OCHO on MOD-Bi, MOD-In, and MOD-BiIn surfaces. Reprinted with permission from [62]. Copyright 2023, Elsevier.

Zhang et al. proposed a unique ZNCS/ZNCO heterostructure (Figure 3a) obtained by vulcanization of ZNCO for positive electrodes of SCs [60]. The resulting flower-like ZNCS/ZNCO composite exhibits several noteworthy advantages, including excellent conductivity, robust structural stability, and enhanced redox reactions. Therefore, the incorporation of oxide heterostructures has a positive impact on the performance of SCs.

In another work, Ju et al. proposed a ternary hollow CuO/ NiO/Co_3_O_4_ heterostructure (Figure 3b) [61]. In this work, core–shell-structured Prussian blue analogs (PBAs) (NiHCC@CuHCC) with Ni-based PBAs (NiHCC) as the core and Cu-based PBAs (CuHCC) as the shell were prepared using the crystal seed method. The enhanced electrochemical performance could benefit from the following characteristics: (1) The hollow structure can provide more active sites, reduce the structural strain, and keep the electrode from collapsing. (2) The electronic structure of CuO/NiO heterojunction can be well tuned and finally facilitate the electron/ion migration [61].

In general, the synergetic effect between various metal oxides in a heterostructure facilitates electron/ion migration. More importantly, heterostructures often combine the advantages of all materials to achieve multiple advantages in various physical and chemical properties [61,63,64].

- Excellent properties for electrocatalysis

The unique features of metal-oxide heterojunctions also confer substantial advantages in electrocatalytic applications. This section will elucidate the merits of metal-oxide heterojunctions in electrocatalysis.

Traditionally, catalysts with high CO_2_ reduction reaction (CO_2_RR) selectivity have relied on weak metal–hydrogen bonds to suppress hydrogen evolution reaction activity [65,66,67]. In recent years, materials derived from metal oxides or sulfides have been found to exhibit better electrocatalytic activity than their corresponding pure metal counterparts [68,69]. Specifically, Bi-based materials such as Bi-MOF, Bi_2_O_3_, or Bi_2_O_2_CO_3_ are commonly used as precursors for efficient electrocatalysts. However, these metal-oxide materials do not enhance the performance or maintain high selectivity [62]. However, indium-based metal-oxide heterojunctions can promote selectivity, especially when indium atoms are introduced at the metal sites of a heterojunction (Figure 3c) [70,71,72].

As shown in Figure 3d, Ye et al. synthesized a material containing partial bimetallic oxides (In_x_Bi_2_-xO_3_) and heterojunctions (In_2_O_3_-Bi_2_O_3_) [62]. In this work, it was shown that heterojunctions can improve performance while maintaining high formate selectivity. This demonstrated that metal-oxide heterostructures have great potential for electrocatalysis. Furthermore, the role of heterojunctions can be also considered as an enlightening contribution in neuromorphic applications.

## 3. Neuromorphic Devices Based on Metal-Oxide Heterojunctions

### 3.1. Biological Synapses and Synaptic Plasticity

Neurons are interconnected by synapses. A synapse specifically connects the axonal terminals of the presynaptic neuron and postsynaptic neuron, thus completing the information transmission between nerve cells [8]. The structure of the human brain is complicated, with ~10^15^ synapses and ~10^11^ neurons [8,73,74].

Synaptic plasticity is an important feature of synapses. It is often considered to be the major cellular mechanism for learning and memory [75]. According to the duration, synaptic plasticity can be divided into short-term plasticity and long-term plasticity. Short-term plasticity has a shorter duration of action, typically tens of milliseconds to minutes. Long-term plasticity, including long-term potentiation (LTP) and long-term depression (LTD), can last much longer, from tens of minutes to even some days [76]. Meanwhile, the conversion between LTP and LTD allows for the easy regulation of synaptic weights [77,78]. In the work of Liu et al., artificial devices with Li-AlO_x_ ion electrolytes were proposed [79], and the short-term potentiation (STP) and LTP emulations of the synaptic weight modulation by the ion electrolyte were emulated based on the electrostatic coupling of Li ions in the electrolyte and electrochemical doping with the metal-oxide In_2_O_3_, respectively [80,81]. After the gate-electrolyte stimulus, the diffusion of the accumulated ions in the electrolyte as the result of the gradient potential leads to the decay of the channel current to the resting level, emulating STP behavior. Electrical stimulus with higher amplitudes or long durations induces electrochemical doping, leading to an LTP behavior of the synaptic weight [9].

Normally, memory is divided into two categories by duration: short-term memory (STM) and long-term memory (LTM). In the process of consolidating LTM, STM processes are responsible for cognition. STM can be converted into LTM. STM can be achieved by the continuous firing of neurons [82]. It is believed that LTM is related to the change in the probability of neurotransmitter release by presynaptic neurons, the change in the sensitivity of postsynaptic neuronal receptors to neurotransmitters, and the change in synaptic structure and synthesis of new proteins [83]. The relationships and properties of LTM and STM need to be further studied. That is why it is important to fabricate devices capable of regulating pre- and post-synapses in synaptic electronics.

Emerging metal-oxide heterojunctions can provide a good opportunity for developing brain-inspired neuromorphic devices. Neuromorphic metal-oxide heterojunction devices can be classified as two-terminal memristors and three-terminal transistors. In the following section, we provide a brief description of the working mechanisms of these different device structures.

### 3.2. Two-Terminal Devices Based on Metal-Oxide Heterojunctions

- Forming conducting filaments

**Figure 4:**
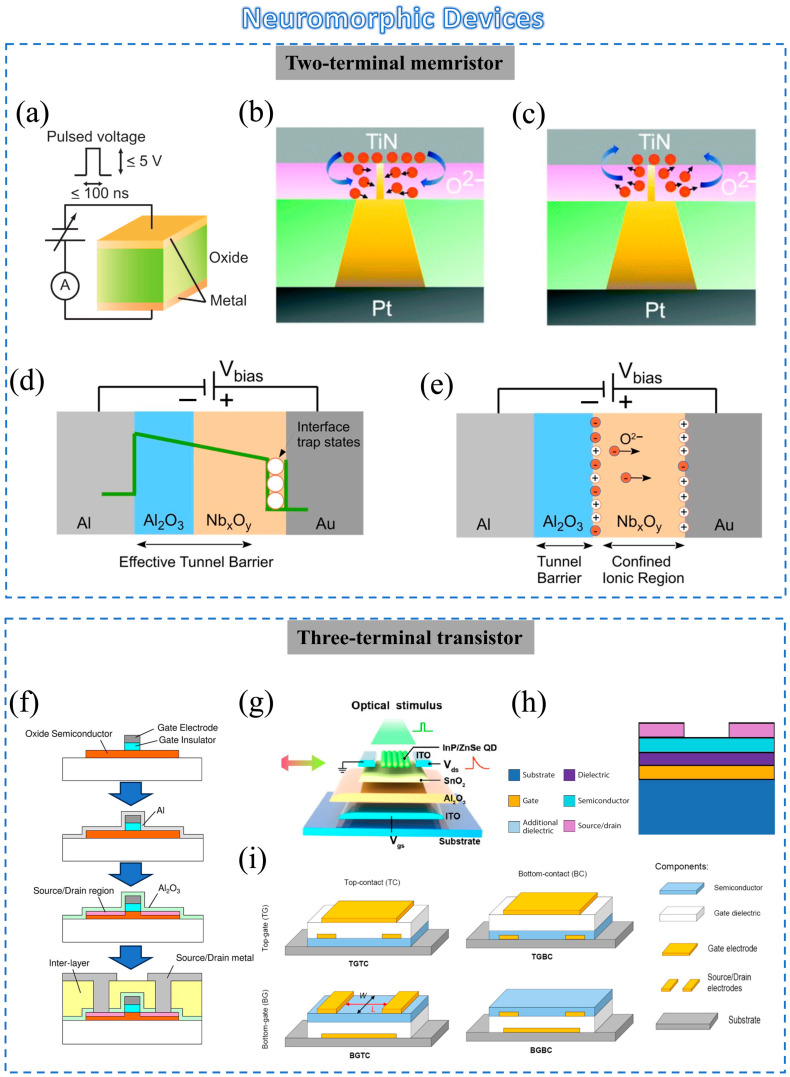
(**a**) Diagram of a resistance random access memory cell with a capacitor-like structure. Reprinted with permission from [84]. Copyright 2008, Elsevier. Schematic of filament (**b**) growth and (**c**) dissolution dynamics. Reprinted with permission from [89]. Copyright 2009, RSC Pub. (**d**,**e**) Two models describing the memristive double-barrier tunnel junctions. (**d**) Simplified cross-sectional view of the memristive tunnel junctions. (**e**) An alternative model to (**d**) [92]. (**f**) Process flow for fabrication of a self-aligned top-gate oxide TFT. Reprinted with permission from [98]. Copyright 2013, John Wiley and Sons. (**g**) Schematic structure of the InP/ZnSe quantum dot (QD)/SnO_2_ hybrid TFT. Reprinted with permission from [27]. Copyright 2022, American Chemical Society. (**h**) Schematic diagrams of the device structures with bottom gate. Reprinted with permission from [20]. Copyright 2015, IEEE. (**i**) Scheme of the four transistor structures: top gate, top contacts (TGTC); top gate, bottom contacts (TGBC); bottom gate, top contacts (BGTC); bottom gate, bottom contacts (BGBC). Reprinted with permission from [99]. Copyright 2020, American Chemical Society.

Two-terminal synaptic devices, also known as memristors, have resistive states that depend heavily on past states, which opens up the possibility of emulating synaptic connections between neurons. The typical device structure is based on a metal-oxide resistive switching layer sandwiched between two metal electrodes [84,85], as shown in Figure 4a. A “SET” voltage switches the device from a high-resistance state to a low-resistance state due to the electrically generated oxygen vacancies (Vo) in the metal-oxide layer. In contrast, a “RESET” voltage switches the device from a low-resistance state to a high-resistance state due to the restoration of the Vo [86,87]. In general, after the SET process, one or more conductive filaments are formed in the oxide layer, while some of the conductive filaments are broken after the RESET process [88].

Moreover, metal-oxide heterojunctions can tune the filament microstructure by controlling the growth and dissolution rates. Wang et al. proposed a resistive switching device by introducing a SiO_2_ ion diffusion limiting layer (DLL) at the TiN/TaO interface (Figure 4b,c) [89]. The device architecture consisted of a multi-layered Pt/TiN/SiO_2_/TaO_x_/Pt stack. The DLL layer led to more linear conductance changes and holds high promise for neuromorphic applications [90].

- Vacancy migration

The relatively high mobility of oxygen-related defects can also be used as an important method for adjusting the synaptic weights in memristors. Oxygen anion migration under an external electric field is one of the main resistive switching phenomena [21]. Dmitri B. Strukov et al. introduced a physical model for Pt/TiO_2_/Pt devices [77,91]. As reported by Hansen et al. in 2015, Al/Al_2_O_3_/Nb_x_O_y_/Au double-barrier memristors were proposed for the optimization of electron tunneling [92] and ion diffusion [89]. Two models used to describe the double-barrier tunnel junctions are shown in Figure 4d,e. This migration enables nuanced, area-dependent resistance modulation. These studies highlight the critical role of vacancy migration in achieving precise control over electrical conductance, laying the groundwork for more stable and efficient artificial synapses [90].

### 3.3. Three-Terminal Devices Based on Metal-Oxide Heterojunctions

While memristors primarily focus on information storage and plasticity, the conductance of neuromorphic field-effect transistors (FETs) can be well controlled through the three terminals [93]. In fact, the gate electrode in a transistor can be viewed as the presynaptic membrane, while the channel layer can be treated as the postsynaptic membrane [94,95,96]. The presynaptic stimulus is a spike input from the gate electrode, and the change in channel conductance is used to emulate the excitatory postsynaptic current [79,93]. Furthermore, synaptic characteristics such as paired-pulse facilitation (PPF), LTP, and LTD can be easily observed in heterostructures for neuromorphic computing [97].

Herein, we categorize metal-oxide FETs based on the device architecture with two types, namely top-gate and bottom-gate FETs, as detailed in the following section.

#### 3.3.1. Bottom-Gate FETs

Bottom-gate FETs have the simplest device structure. For example, the production of the BGBC structure shown in Figure 4h just needs one lithographic step, which makes the device fabrication relatively quick and cheap [20]. The bottom-gated three-terminal FETs proposed by Rehman et al. are auspicious candidates for the emulation of biological functions in realizing proficient neuromorphic computing systems [100]. These devices exploit the hysteresis effect in the transfer curves of that kind of FET to explore excitatory/inhibitory post-synaptic currents (EPSCs/IPSCs), LTP, LTD, spike timing/amplitude-dependent plasticity (STDP/SADP), and PPF.

As a special kind of FET, TFTs were first fabricated in 1962 by Paul K. Weimer, and in 1979, the first functional TFT was successfully demonstrated [101,102]. Oxide-based TFTs were first reported in 1964 by Klasens et al., and then the most famous indium gallium zinc oxide TFT was proposed by Nomura et al. in 2004 [103,104]. Oxide TFTs have obvious advantages: high mobility, good stability, nice chemical resistance in liquids, high transparency, and ease of processing [94,105,106].

In some research, managing vacancies has proved important for improving the performance and stability of oxide TFTs, particularly in heterojunction configurations. Liang et al. [27]. proposed a structure of InP/ZnSe QD/SnO_2_ hybrid TFTs using full printing technology. In this structure (Figure 4g), the band alignment near the heterojunction resulted in the effective separation of photogenerated electrons and holes, which resulted in a spectrally dependent response in photonic synaptic devices. Using both the high photosensitivity of InP/ZnSe QDs and the high electron mobility of SnO_2_, the devices could well emulate key synaptic behaviors such as EPSC, STP, LTP, and PPF. An artificial vision system was finally demonstrated with enhanced image recognition and processing efficacy. These findings underscore the viability of employing such heterojunction devices for optoelectronic synapses and also offer a scalable fabrication strategy for advanced artificial vision systems [27].

#### 3.3.2. Top-Gate FETs

As for the top-gate structure, the fabrication process involves the channel structure, followed by the gate dielectric and gate electrode (Figure 4f) [98]. In this structure, the current flows effectively at the top interface of the semiconductor. The semiconductor layer is encapsulated by dielectric and gate electrodes, protecting it from damage in subsequent processing steps.

However, at the same time, the semiconductor may be also affected by thermal cycling and solvents in subsequent process steps, potentially deteriorating the transistor performance. When a sintering step is employed, a bottom-gate structure may be more suitable. This is because subsequent sintering steps can degrade the semiconductor performance in top-gate transistors. Although top-gate and bottom-gate structures have their own exquisite manufacturing technology, they all play an important role in neuromorphic applications [107].

## 4. Neuromorphic Applications

In the actual activity of life, the sensory nervous system is formed by various neural networks and circuits connected by synapses with neurons as the basic unit. Neuromorphic devices with metal-oxide heterojunction have attracted increasing interest and become a hot topic in recent years. In the following sections, we summarize the recent progress for these neuromorphic devices in visual, tactile, and nociceptive systems.

### 4.1. Neuromorphic Visual System

**Figure 5:**
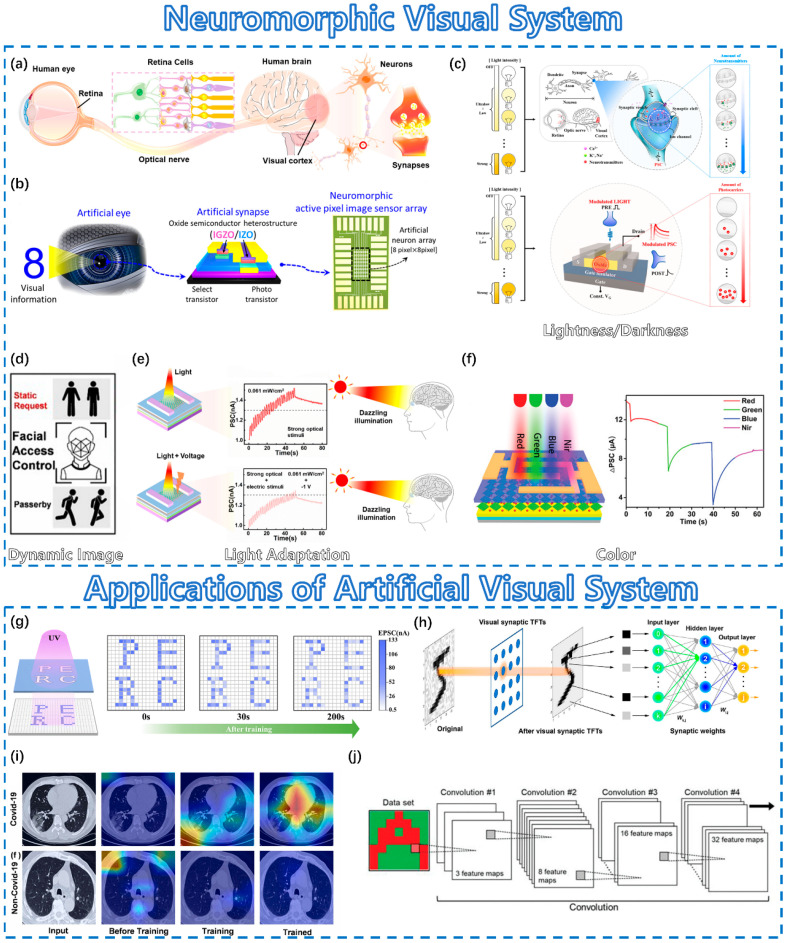
Neuromorphic visual system. (**a**) Schematic diagram of the human visual system. Reprinted with permission from [109]. Copyright 2022, American Chemical Society. (**b**) Schematic diagram of artificial visual system. Reprinted with permission from [112]. Copyright 2021, American Chemical Society. Some typical visual features: (**c**) Lightness and darkness. Reprinted with permission from [109]. Copyright 2022, American Chemical Society. (**d**) Dynamic image [113]. (**e**) Light adaptation. Reprinted with permission from [114]. Copyright 2022, Elsevier. (**f**) Color perception. Reprinted with permission from [115]. Copyright 2021, John Wiley and Sons. Several applications of artificial visual systems: (**g**) Visual information learning and memory processing. Reprinted with permission from [27]. Copyright 2022, American Chemical Society. (**h**) Image preprocessing. Reprinted with permission from [79]. Copyright 2022, Elsevier. (**i**) Image recognition. Reprinted with permission from [116]. Copyright 2022, John Wiley and Sons. (**j**) Color pattern recognition. Reprinted with permission from [117]. Copyright 2023, Royal Society of Chemistry.

The visual nervous system is an essential way for humans to acquire external information, more than 80% of which is obtained by vision [108]. In the nervous system, when incident light enters the eyeball, optic nerve fibers in the retina convert external signals into biological signals and transmit action potentials to the visual cortex in the brain to form vision, as shown in Figure 5a [109,110,111]. Figure 5b illustrates a method of emulating the human visual nervous system using neuromorphic devices [112].

The visual system under lightness/darkness conditions can be successfully emulated as shown in Figure 5c. When a signal is transmitted to the terminal of the presynaptic neuron, the electrical signal is converted into a chemical signal and accompanied by the release of neurotransmitters, which can bind to the receptors of the postsynaptic neuron to complete the signal transmission between neurons. When artificial synapses are stimulated by external light, the number of photocarriers can be increased to tune the device conductance, which is similar to the function of neurotransmitters in the actual nervous system. Therefore, the photoresponse of artificial synapses is weaker in the dark than in sufficient light, which is very consistent with human visual neural behaviors [113]. This helps neuromorphic devices to capture both static and dynamic images, as shown in Figure 5d [114]. Under bright light, a photoreceptor with rod and cone cells will adapt to external light and then produce the appropriate level of electrical spikes according to the ambient light intensity, which is known as light adaptation [118,119]. By introducing a negative voltage pulse, a neuromorphic device can adapt to light and avoid possible damage from strong light, as shown in Figure 5e [109]. At the same time, a neural network can reduce the response of nerve impulses near the target, and color recognition can be demonstrated [120]. Different neuromorphic devices can be designed to realize light absorption for the recognition of different colors, as shown in Figure 5f [115,116,121,122]. Different visual functions can be successfully demonstrated, such as visual information learning/memory, image preprocessing, and color pattern recognition [27,79,116,117].

### 4.2. Neuromorphic Tactile System

**Figure 6:**
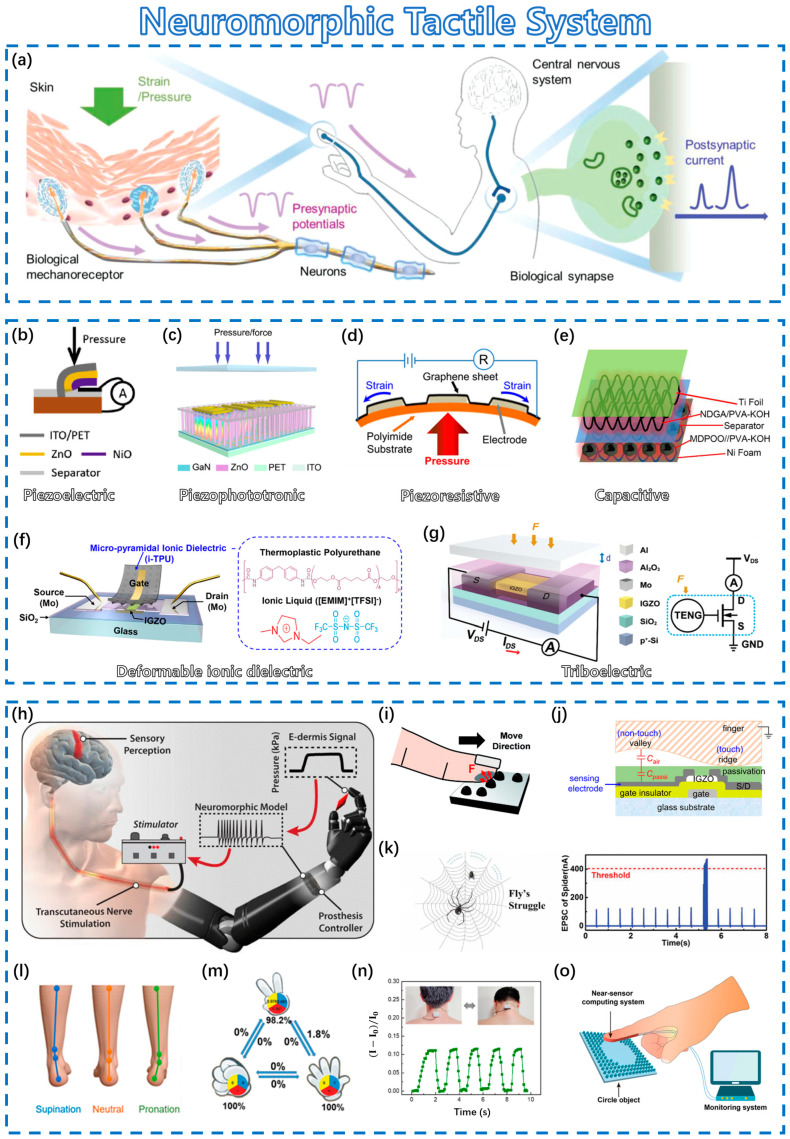
Neuromorphic tactile system. (**a**) Schematic diagram of the human tactile nervous system. Reprinted with permission from [24]. Copyright 2019, John Wiley and Sons. Sensing mechanisms: (**b**) Piezoelectric. Reprinted with permission from [125]. Copyright 2020, Elsevier. (**c**) Piezophototronic. Reprinted with permission from [127]. Copyright 2019, Elsevier. (**d**) Piezoresistive. Reprinted with permission from [147]. Copyright 2020, John Wiley and Sons. (**e**) Capacitive. Reprinted with permission from [148]. Copyright 2018, American Chemical Society. (**f**) Piezocapacitive. Reprinted with permission from [149]. Copyright 2020, John Wiley and Sons. (**g**) Triboelectric Reprinted with permission from [150]. Copyright 2017, American Chemical Society. Applications of neuromorphic tactile systems: (**h**) Prosthesis with tactile sensor feedback. Reprinted with permission from [131]. Copyright 2019, IEEE. (**i**) Touching convex braille numbers. Reprinted with permission from [132]. Copyright 2012, Royal Society of Chemistry. (**j**) Touch-fingerprint sensor. Reprinted with permission from [133]. Copyright 2018, John Wiley and Sons. (**k**) Mechanical sensory mechanism. Reprinted with permission from [134]. Copyright 2020, IEEE. (**l**) Physiological signal detection. Reprinted with permission from [135]. Copyright 2018, American Chemical Society. (**m**) Recognition of three gestures. Reprinted with permission from [136]. Copyright 2019, IEEE. (**n**) Monitoring human motions. Reprinted with permission from [137]. Copyright 2023, American Chemical Society. (**o**) Near-sensor analog computing system. Reprinted with permission from [151]. Copyright 2022, John Wiley and Sons.

Skin, as the largest sensory organ in the human body, plays an important role in the actual interaction process. It is embedded with countless tactile receptors, helping the body to complete a series of complex physical interactions, such as snatching, sliding, and the judgment of object properties [123]. The basic principle of the human tactile nervous system is shown in Figure 6a. An artificial tactile nervous system may be a powerful way to realize the future of bionic robots. So far, various ways to implement these systems have been reported, including the use of piezoelectric, piezophototronic, piezoresistive, capacitive, deformable ionic dielectric, and triboelectric materials, as detailed below.

(i) Piezoelectric materials generate an electrical potential when the pressure/stress changes, which achieves the conversion of mechanical signals to electrical signals, as shown in Figure 6b [124,125]. (ii) The piezophototronic effect is that the interface band structure is effectively modulated by a piezoelectric polarization charge caused by external stress [126]. A piezophototronic material can be used in a light-emitting diode array of pixels, resulting in a nice pressure distribution, as shown in Figure 6c [127]. (iii) The resistance of a piezoresistive material will change with external pressure or stress. Consequently, piezoresistive materials could be deployed as tactile receptors to detect mechanical stress or pressure, as shown in Figure 6d. This change in resistance largely comes from the variation in the conductive path or structure of the material itself. Wang et al. fabricated a carbon nanotube paper film (CNTF)/stress-induced square frustum structure (SSFS) pressure sensor with ultra-sensitive and wide-range flexible performance. An external effect will change the inner conductive path of the sensor, which is composed of contacts between interdigital electrodes (IDEs) and fibers inside the CNTF as well as contacts between fibers themselves [128]. Cheng et al. developed sensors based on Au/graphene composite films (AGCFs) with hierarchical cracks. By using electrical resistance varying with the change in crack number and areas, the sensing performance presents high sensitivity, excellent linearity, and adjustability due to tuning pattern regulation [129]. (iv) For capacitive tactile receptors, external effort usually causes a change in the thickness of the dielectric layer, which affects the conductive value, mimicking skin, as shown in Figure 6e. Recently, there has also been great interest in utilizing 4D printing devices to fabricate capacitors to be used in physiological monitoring sensors [130]. (v) In a deformable ionic dielectric, external pressure will effectively force ions accumulated at the interface between the semiconductor and dielectric to influence the current level, as shown in Figure 6f. (vi) As for triboelectric receptors, the principal mechanism is the triboelectric nanogenerator (TENG). In devices, the variation in two layers in the TENG will cause the motion of electrons to modulate the gate voltage, as shown in Figure 6g. Tactile devices with the various principles above have been developed, and their functions in neuromorphic tactile systems are endless.

Figure 6h shows a prosthetic hand with tactile sensors that can transform mechanic signals into nervous signals. Although only three tactile sensors are integrated per finger, they can still help amputees feel pain and distinguish shapes [131]. As shown in Figure 6i, tactile devices with high sensitivity to pressure can be utilized to help blind people learn braille or recreate the sense of touch [132]. Since a capacitive tactile device is highly sensitive to distance, fingerprint recognition was successfully realized, as shown in Figure 6j [133]. In addition, the typical functions of tactile neurons can be emulated to perceive dynamic pressures with different intensities, sizes, and frequencies, as shown in Figure 6k [134]. Tactile devices have many promising applications, as shown in Figure 6l for physiological signal detection [135], Figure 6m for recognition of three gestures [136], Figure 6n for monitoring human motions [137], and Figure 6o for near-sensor analog computing [24].

### 4.3. Neuromorphic Nociceptive System

**Figure 7:**
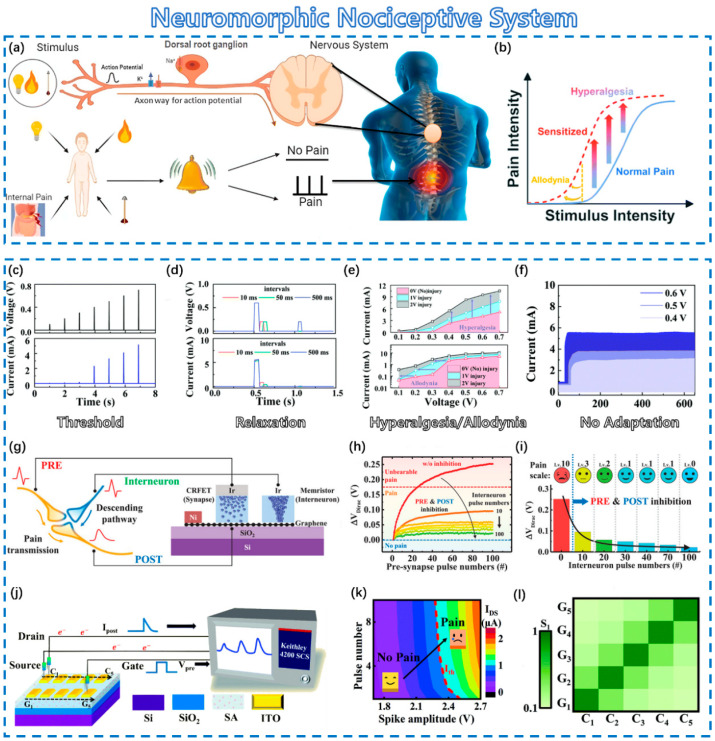
Neuromorphic nociceptive system. (**a**) Schematic diagram of human nociceptive nervous system. Reprinted with permission from [139]. Copyright 2009, Royal Society of Chemistry. (**b**) Characteristics of nociceptive nervous system: (**c**) threshold, (**d**) relaxation, (**e**) allodynia/hyperalgesia, (**f**) no adaptation. Reprinted with permission from [146]. Copyright 2012, Royal Society of Chemistry. Artificial pain modulation system: (**g**) schematic diagram, (**h**) pain inhibition in the artificial tactile nervous system, and (**i**) inhibition abilities. Reprinted with permission from [152]. Copyright 2022, John Wiley and Sons. Graded pain perception: (**j**) oxide transistor arrays and test system, (**k**) evaluations of pain-perception behaviors, and (**l**) degree of pain sensitivity for different gate–channel combinations. Reprinted with permission from [153]. Copyright 2009, Royal Society of Chemistry.

Tactile perception is one of the primary methods by which the human body coordinates and interacts with its surroundings. At the same time, touch operation is also the primary way of human–computer interaction. Human skin is covered with different types of mechanoreceptors. When the skin senses changes in pressure, temperature, or humidity in the external environment, it sends out tiny electrical signals that are transmitted to the brain with nerve fibers, which in turn produce the sense of touch [138]. The combination of tactile sensors and synaptic devices provides a solution for implementing neuromorphic functions for strain pattern recognition and processing [24]. As critical sensory components in the nervous system, nociceptors can recognize noxious stimuli collected from tactile sensory signals and transmit signals to the central nervous system, enabling a response that avoids damage to the body, as shown in Figure 7a [139]. Figure 7b shows the characteristics of the nociceptive nervous system. There are several basic characteristics in the nociceptive system [140,141,142,143,144,145]: (i) Threshold behavior means that a significant response appears until the external stimulus approaches or even exceeds the threshold value, as shown in Figure 7c. (ii) Relaxation behavior means that nociceptors can maintain an extremely high response after the removal of stimuli, as shown in Figure 7d. (iii) Hyperalgesia and allodynia behaviors indicate an enhanced response to noxious stimuli is generated even below the stimulus threshold, as shown in Figure 7e. (iv) Adaption means that nociceptors will not respond to dangerous stimuli, as shown in Figure 7f [146].

Fu et al. proposed a kind of charge-regulated FET (CRFET) to realize an artificial pain-perceptual system that adopted graphene as a channel and Gd_x_O_y_/Al_x_O_y_ as dielectric layers to provide opposite polarities of oxide charges [152]. The Gd_x_O_y_ dielectric layer containing positive oxide charges plays an important role in the IPSC/EPSC behaviors. However, the neuromorphic behaviors would be opposite for the Al_x_O_y_ dielectric layer with negative oxide charges. An artificial pain modulation system (PMS) was proposed based on the above behaviors [152]. Figure 7g shows biological PMS and artificial PMS. The enhanced conductivity of the memristor can increase the voltage drop of the channel and effectively suppress the amplitude of the signal from the gate, as shown in Figure 7h. Figure 7i shows the ability of the pre-synapse to inhibit both pre- and post-stimulus signals.

For array networks, Li et al. utilized sodium alginate (SA) as a common neurotransmitter layer to fabricate a 5 × 5 ionotronic junctionless indium-tin-oxide transistor array for realizing pain-perception and threshold-modulation functions [153]. Figure 7j shows the oxide transistor array and test system. Pain is also recognized by defining a threshold current, as shown in Figure 7k,l. A coplanar–multiterminal transistor array is very suitable for not only realizing the nociceptor networks but also avoiding complex architectures with multiple layers, interconnects, or other complex components. Moreover, this particular structure also has the ability to modulate synaptic weights according to spatial orientation, through which orientation-dependent spike timing plasticity learning rules can be successfully implemented. Such devices provide a viable method for building spatiotemporally correlated neuromorphic systems [154]. This device can provide a novel approach for achieving praiseworthy pain-perceptual functions and may also create a new opportunity for oxide transistor arrays in future multifunctional robotics and auxiliary equipment.

## 5. Conclusions and Perspectives

This review investigates neuromorphic devices based on metal-oxide heterostructures, encompassing their fabrication, properties, and applications. Initially, we discuss various preparation methodologies for metal oxides, including both bottom-up and top-down approaches. Subsequently, the fabrication methods and properties of metal-oxide heterostructures are elaborated. Further, the review delves into different neuromorphic device architectures with metal-oxide heterostructures, including two-terminal memristors as well as three-terminal transistors. More importantly, we present the recent progress in their neuromorphic applications, including in visual, tactile, and nociceptive systems. Metal-oxide heterostructures have several advantages, such as low power consumption, high stability, and excellent electrical performance. However, there are still several challenges that cannot be overlooked. Due to the nonlinear and dynamic nature of metal-oxide heterostructures, there are various aspects that need to be considered when constructing metal-oxide heterostructures, such as determining how to obtain a combination of the excellent properties of the two materials and make them outstanding while at the same time keeping other poor properties of the two materials that are not conducive to the performance of the device from becoming outstanding. The integration of metal-oxide-based neuromorphic devices with existing silicon-based electronic devices still faces compatibility and interconnectivity challenges, as the fabrication of metal-oxide heterostructures is incompatible with conventional CMOS fabrication techniques. As far as bionic sensing systems are concerned, most of the current research has only been directed towards systems with a single sensing capability through the integration of sensors and synaptic devices, e.g., vision and haptics.

In light of these drawbacks, several remedial strategies can be considered. Firstly, data analytics based on a machine learning approach could be employed to accurately predict material properties and device performance. Secondly, researchers could investigate metal-oxide materials and device structures that are compatible with existing CMOS processes. Also, researchers could develop simplified manufacturing processes to reduce manufacturing steps and costs. Then, they could design flexible interface technologies for the seamless integration of metal-oxide devices with silicon-based circuits. Also, they could develop modular designs so that components from different technologies can be easily integrated. Finally, new micro- and nanofabrication techniques for reducing device variability and leakage on small scales should be researched and developed. Also, 3D integration techniques for increasing device density without sacrificing performance should be explored.

Nevertheless, neuromorphic devices based on metal-oxide heterojunctions integrating sensing, storage, and computing are urgently needed for the booming fields of artificial intelligence and machine learning. Therefore, research on synaptic devices based on metal-oxide heterojunctions is an exciting topic in the age of intelligence. From the perspective of future applications, especially in the field of self-driving cars, smart sensors, and personalized medicine, the functions of metal-oxide heterojunction neuromorphic devices in synapse emulation, image sensing, and preprocessing will be further developed through rational material selection and device structure design. In conclusion, the field of metal-oxide heterostructures for neuromorphic applications holds great promise for revolutionizing various aspects of computing and artificial intelligence. Continued research and development in this area are expected to yield groundbreaking advancements in the coming years.
